# Gender differences in geriatric depressive symptoms in urban China: the role of ADL and sensory and communication abilities

**DOI:** 10.3389/fpsyt.2024.1344785

**Published:** 2024-02-28

**Authors:** Ling Guo, Mingwang Fang, Lingying Wang, Li Liu, Chunxia He, Xiumei Zhou, Yi Lu, Xiuying Hu

**Affiliations:** ^1^ Innovation Center of Nursing Research and Nursing Key Laboratory of Sichuan Province, West China Hospital, Sichuan University/West China School of Nursing, Sichuan University/Institute for Disaster Management and reconstruction, Sichuan University, ChengDu, China; ^2^ West China Hospital, Sichuan University, Cheng Du, China

**Keywords:** ADL, depressive symptoms, gender difference, sensory and communication abilities, urban China

## Abstract

**Objectives:**

ADL and Sensory and Communication Abilities are important indicators of the quality of life of the elderly which are significant determinants of health, particularly in developing countries. The present cross-sectional study investigated effect of ADL and Sensory and Communication Abilities on depressive symptoms, as well as the the role of gender in these effects.

**Design:**

This is a cross-sectional study.

**Setting:**

A nationally representative cross-sectional survey among the Chinese population aged 60 years and over.

**Participants:**

A total of 163296 females and 148724 males aged 65 and over in 2019 in urban China.

**Outcome measures:**

Prevalence, risk factors and gender differences in geriatric depressive symptoms among urban elderly.

**Results:**

Approximately 95.69% of the participants had depressive symptoms according to the CESD-10, with no statistically significant gender difference of 52.15% in females and 47.85% in males. Logistic regression findings suggest that geriatric depressive symptoms are significantly associated with the lack of eldercare (OR=2.427, female; OR=1.426, male), living alone(OR= 1.430, female; OR= 1.179, male), ADL dysfunction (OR=1.528, female; OR=1.246, male), and impaired sensory and communication ability (OR=1.338, female; OR=1.185, male) among both female and male participants. Remarkably, geriatric depressive symptoms are only significantly associated with age (≥75, OR = 1.327), marital status (unmarried, OR=1.598), the number of children (no children, OR=2.271), and the living arrangement (living alone, OR= 1.430) among female participants.

**Conclusion:**

Significant gender differences in these associations were found for living alone, ADL dysfunction and impaired sensory and communication ability. Moreover, the study emphasized that the gender difference exists in terms of geriatric depression in urban China. Females are more likely to experience depressive than males with the same circumstances.

## Introduction

Aging is a global phenomenon and a complex biological process of all living beings that bring about a wide variety of changes. The total number of people aged 65 and above increased from 697.97 million in 2019 to 723.48 million in 2020. Among them, the number of women was 398.55 million, and that of men was 32.49 million, with women outnumbering men by 73.61 million (1). By 2030, 1 in 6 people in the world will be aged 60 years or over. At this time the share of the population aged 60 years and over will increase from 1 billion in 2020 to 1.4 billion. With advances in medicine helping more people to live longer lives, more than 1 in 5 people will be 60 years or older by 2050, according to a new report released by the WHO for the International Day of Older Persons (2). By 2050, the world’s population of people aged 60 years and older will double (2.1 billion). The number of persons aged 80 years or older is expected to triple between 2020 and 2050 to reach 426 million (3). China has the largest population of elderly people, with whose number of people over the age of 60 reaching 168.86 million in 2020, about 23.34% of the total number of elderly people in the world. Population data in 2021 shows that the number of elderly people aged 65 years and above is up to 20.56 million in China, which has exceeded 200 million. And 267.36 million people aged 60 and above (4).

The pace of population aging is much faster than in the past. In 2050, 80% of older people will be living in low- and middle-income countries. All countries face major challenges to the physical and mental health of older people, especially in developing countries and backward countries. Mental health and well-being are as important in older age as at any other time of life. Mental and neurological disorders among older adults account for 6.6% of the total disability (DALYs) for this age group. Approximately 15% of adults aged 60 and over suffer from a mental disorder (5). Depression is a common mental disorder and a serious public health problem. An estimated 3.8% of the global population suffered from depression, including 5.0% among adults and 5.7% among people over 60 years, which is a leading cause of disability worldwide and a major contributor to the overall global burden of disease. Studies of depression in older adults dating back to the 1980s (6).

According to a World Health Organization report published in 2021, more women are affected by depression than men. Some studies have also shown that epidemiological gender differences in depression are well characterized (7–11). Women are more likely to suffer from depression throughout their lives and are twice as prevalent as men (12). Luppa found a prevalence of depression of 4.0% to 10.3% among women over 75 years and 2.8% to 6.9% among men of the same age. At the same time, the prevalence increased slightly in women over 85 years, but not in men (13).

This gender gap may be partly explained by the fact that men have lower life expectancy than women considering at the macro or societal level, leading to the widowed and altered social environment in old age for women. At the same time, women and girls are more vulnerable to violence and structural gender inequalities (12). However, depression may be related to many factors, not only biological, psychological, and self-perception factors, but also complex sociocultural factors, such as social role, social participation, and social support (14).

As an important component of social support that elderly people receive from their partners or family members, living arrangements has commonly been viewed as a risk factor for depression.

in the elderly.Living arrangements for older adults with mental illness can have a significant impact on the quality of life for both the individual and their loved ones. A key factor in determining the best living arrangement for elderly people with mental illness is understanding the different options and determining which one will be the most beneficial. Generally, living arrangements could refer to a type of personal connection, in which a person’s family relationship and non-family relationships are connected with other people he lives with (15). The elderly have a variety of living arrangements, including living with their spouses/children/brothers and sisters/other relatives/pension agency and living alone. Several studies have shown that living arrangements are related to depression in older adults. Older adults who live alone are more likely to be depressed than those who live with others. Gender differences in the effect of living arrangements on the risk of depression among older adults are stark.

This article aims to explore the gender differences in geriatric depressive symptoms in urban China, with a specific focus on the role of activities of daily living (ADL) and sensory and communication abilities. ADL refers to the basic self-care tasks necessary for an individual’s functional independence, including personal hygiene, dressing, eating, toileting, transferring, and continence. Sensory and communication abilities encompass the sensory perception and communication skills required for social engagement and functional participation. Sensory and communication measures have been linked to the mental health of individuals (16, 17). The interaction between stimuli from the environment, including sound, light, smell, and other sensations, and the individual’s ability to articulate, perceive, and process those stimuli affects their mental health. In some cases, these measures can play a role in behavioral and mental health problems, while in other cases they can be used to help individuals improve their overall mental well being. Sensory and communication deficits are common among older adults, and they can increase the risk of developing mental illnesses such as depression and dementia. Research has shown that sensory and communication deficits can interfere with a person’s ability to interact with their environment, leading to increased stress and isolation. This can put older adults at a greater risk of developing mood and cognitive disorders. Sensory and communication deficits can be caused by a variety of factors, including age-related changes in hearing, vision, or speech. It may also be caused by side effects from certain medications, or certain neurological conditions. Regardless of the cause, sensory and communication deficits can impede a person’s ability to interact with their environment and can lead to feelings of loneliness and depression.

The significance of this research lies in the need to comprehensively understand the factors contributing to gender differences in geriatric depressive symptoms. While previous studies have identified gender disparities in depression, few have explored the specific role of ADL and sensory and communication abilities in urban Chinese older adults. By examining this relationship, we can gain valuable insights into the unique challenges faced by elderly individuals in urban China and inform targeted interventions and support systems. Identifying the factors that contribute to depressive symptoms in older adults, particularly within the context of gender differences, has important implications for healthcare providers, policymakers, and society as a whole. By elucidating the role of ADL and sensory and communication abilities, we can develop more effective strategies to prevent, detect, and treat depressive symptoms in geriatric populations. Additionally, this research can aid in developing tailored interventions that address the specific needs and challenges faced by elderly individuals, promoting their mental well-being and overall quality of life.

We conducted this research to explore the gender differences in geriatric depressive symptoms in urban communities in Sichuan province of China, and further examined the influence of living arrangements and sensory and communication on depressive symptoms. It was hypothesized that elderly individuals with impaired ADL ability and sensory and communication ability would be more likely to experience depressive symptoms and this relationship would be different for specific types of participant characteristics. In addition, gender would moderate the relationship between ADL, sensory and communication ability and depressive symptoms.

Overall, this study seeks to fill a critical gap in the literature by examining the role of ADL and sensory and communication abilities in the gender differences observed in geriatric depressive symptoms in urban China. The findings will contribute to a better understanding of the complex factors influencing depression in older adults, ultimately leading to more targeted interventions and improved mental health outcomes for this vulnerable population.

## Strengths and limitations of this study

### Strengths

#### Sample size

The study used the nationally representative data includes a substantial sample size of 312020 elderly individuals from urban China to implement the cross-country comparisons and collection of much more information about the role of ADL and sensory and communication abilities in exploring the development of depressive symptoms in urban China. This large sample enhances the reliability and generalizability of the findings to the broader population.

#### Comprehensive approach

The article takes a multidimensional approach by considering various factors such as gender, ADL performance, sensory and communication skills. This comprehensive analysis provides a more nuanced understanding of the relationship between these variables and geriatric depressive symptoms.

#### Statistical analysis

The researchers employ sound statistical methodologies, such as regression analysis and multiple mediation models, to analyze the data. These robust statistical techniques contribute to the validity and reliability of the results.

### Limitations to address

#### Sampling bias

The study primarily focuses on elderly individuals from urban areas in China, potentially introducing sampling bias. The findings may not be representative of other populations, such as rural areas or different cultural contexts. Future research should aim for a more diverse sample to enhance the external validity.

#### Cross-sectional design

The study adopts a cross-sectional design in 2019, limiting the ability to establish causality. Longitudinal studies could provide further insights into the directional relationships between gender, functional abilities, and depressive symptoms. Future research should consider a longitudinal approach and incorporate objective measures to strengthen the validity of the findings.

#### Self-reported measures

The study relies on self-reported measures to assess geriatric depressive symptoms, ADL performance, and sensory and communication abilities. This approach may introduce response bias and subjectivity. Including objective measures, such as clinical diagnoses and performance-based assessments, would strengthen the validity of the findings.

## Materials and methods

### Study design

This study is based on the National Health Commission of the People’s Republic of China (Health Commission of Sichuan Provincial of the People’s Republic of China), a nationally representative cross-sectional survey among the Chinese population aged 60 years and over. A quantitative approach was employed to collect and analyze data from elderly participants.

### Setting and participants

Respondents’ detailed information were collected by face-to-face interviews in urban and rural communities, including the demographic characteristics, socioeconomic status, health status and living style. The data of this study were derived from the survey in 2019, and the inclusion criteria were: (1) age ≥ 60, (2) living in urban communities of Sichuan Province, (3) needing Care services, and (4) being able to participate in research, participants with severe mental and cognitive impairment had been excluded from the original data. Thus, the number of respondents eligible for our analysis dropped to 312020 elderly.

### Procedure

Once the participants were identified and recruited, data collection took place through face-to-face interviews conducted by trained researchers. The interviews were conducted in Mandarin or the local dialect, depending on the participant’s preference and comprehension.

### Assessment and measurements

#### Depressive symptoms

In the study, the 10-item version of the Center for Epidemiological Studies Depression Scale (CESD-10) was used to evaluate depressive symptoms, which was developed as a modification of the CESD-20 scale by Andresen (18). The time frame for the CESD-10 questions referred to the latest week. Each item ranged from 0 to 3 points, with answers to assessments were rated on four levels: varying from’rarely or none of the time (0–1 day)’to’most or all of the time (5–7 days)’, where positive items were reverse scored. The total scores ranged from 0 to 30, with a higher score indicating a higher level of depressive symptoms.CESD-10 has shown good validity and reliability in the Chinese population. A cut-off point of 10 was used to generate the binary depressive symptom variable since some previous validation studies among Chinese older adults showed that a cut-off point of 10 provides the optimal threshold to identify clinically significant depressive symptoms. Participants who scored more than 10 were classified as depressed (19).

#### Living arrangements

The instrument of living arrangement contained two items: living with others (including spouse\children\brothers and sisters\other relatives\pension agency, yes or no), and living alone (yes or no).

#### Health ability assessment

In this study, the respondents’ health ability assessments were assessed by the “Ability Assessment of the Older Adults” (MZ/T039-2013) which was developed by the Ministry of Civil Affairs of the People’s Republic of China. This assessment tool has shown good reliability and validity in the Chinese population, with a Cronbach’s α score of 0.889. The assessment contains two parts: “activities of daily living scale (ADL)” and “sensory and communication skills scale”.

#### ADL

Activities of daily living (ADL) limitations indicate any self-reported difficulty in any of the following six activities of daily living: controlling urination and defecation, bathing/showering, using the toilet, getting into or out of bed, dressing, or eating. The four response options were: 1=“no difficulty, 2=“ difficulty but can still do it, 3 = “difficulty and need help, 4 = “unable to do.” It was dichotomized as “ADL-unimpaired” vs. “ADL-impaired.” “ADL-impaired” was defined as “have difficulty and need help” or “unable to do” in any item.

#### Sensory and communication skills

The sensory and communication skills scale involved the level of sound, light, smell, communication and other sensations, and the individual’s ability to articulate, perceive, and process those stimuli affects mental health. The four response options were: 1= “no difficulty, 2= “ a little difficulty”, 3=“very difficultly”, 4=“unable to do”. It was also dichotomized as “unimpaired” vs. “impaired”. “Sensory and communication unimpaired” was defined as “no difficulty” in all levels of sensory and communication.

#### Socio-demographic characteristics

Sociod-emographic variables included in the analysis were age (65-74 years, ≥75 years), gender (female,male), marital status [married or unmarried(divorced,widowed, never married)], nationality (Han, others), the number of children(have children, no children), and eldercare status (have eldercare, no eldercare).

#### Socio-economic status

Educational level was used to determine Socio-Economic status(SES) which was classified as ‘primary school and below’ and ‘high school and above’.

#### Patient and public involvement

The study did not involve patients. The results of the survey are disseminated to the public through websites of the public county councils. Informed consent was obtained from all participants prior to their participation in the study. Confidentiality and privacy of the participants’ information were assured throughout the research process.

### Statistical analysis

All the data in this study were conducted using STATA version 16.0 (StataCorp LLC, Lakeway TX, USA) (20, 21), and SPSS version 25.0 (IBM, Armonk, NY, USA). Data were expressed as percentages and proportions of categorical values. First, we described the basic characteristics of the data, and the format of categorical variables was n (%). Then, we conducted binary logistic regression to evaluate the factors affecting depressive symptoms. Finally, we conducted a t-test analysis to assess the interaction between gender and these influencing factors. The results were expressed as OR values and 95% CI. In all the results, p<0.05 was used as the criterion for statistically significant differences.

## Results

### Demographic characteristics

**Table 1** reports sociology-demographic characteristics of the participants by genders. Out of 312020 urban participants 163296(52.34%) are females and 148724(47.66%) are males aged 65 and over.Most participants had lower educational level (elementary school and below), with significantly more females in the low-level group. Approximately 95.69% of the participants had depressive symptoms according to the CESD-10, with no statistically significant gender difference of 52.15% in females and 47.85% in males.

**Table 1:** Distribution and prevalence of depressive symptoms by gender according to participant characteristics.

Variable	Full Samples		*P*	Female		*P*	Male		*P*
	Not Depressed (n=13435)	Depressed (n=298585)		Not Depressed (n=7569)	Depressed (n=155727)		Not Depressed (n=5866)	Depressed (n=142858)	
Age (years)			<0.001			<0.001			<0.001
65-74	6355 (47.30)	180606 (60.49)		3540 (46.77)	93076 (59.77)		2815 (47.99)	87530 (61.27)	
≥75	7080 (52.70)	117979 (39.51)		4029 (53.23)	62651 (40.23)		3051 (52.01)	55328 (38.73)	
Marital status			<0.001			<0.001			<0.001
Married	8545 (63.60)	212251 (71.09)		4305 (56.88)	100433 (64.49)		4240 (72.28)	111818 (78.27)	
Unmarried	4890 (36.40)	86334 (28.91)		3264 (43.12)	55294 (35.51)		1626 (27.72)	31040 (21.73)	
Nationality			<0.001			0.003			0.001
Han	13386 (99.64)	296544 (99.32)		7537 (99.58)	154603 (99.28)		5849 (99.71)	141941 (99.36)	
Others	49 (0.36)	2041 (0.68)		32 (0.42)	1124 (0.72)		17 (0.29)	917 (0.64)	
Education			<0.001			0.971			<0.001
Elementary school and below	11739 (87.38)	256547 (85.92)		6961 (91.97)	143189 (91.95)		4778 (81.45)	113358 (79.35)	
Middle school and above	1696 (12.62)	42038 (14.08)		608 (8.03)	12538 (8.05)		1088 (18.55)	29500 (20.65)	
The number of children			<0.001			0.507			<0.001
Have children	12709 (94.60)	284961 (95.44)		7332 (96.87)	151069 (97.01)		5377 (91.66)	133892 (93.72)	
No children	726 (5.40)	13624 (4.56)		237 (3.13)	4658 (2.99)		489 (8.34)	8966 (6.28)	
Eldercare status			<0.001			<0.001			<0.001
Have eldercare	12656 (94.20)	276736 (92.68)		7134 (94.25)	144723 (92.93)		5522 (94.14)	132013 (92.41)	
No eldercare	779 (5.80)	21849 (7.32)		435 (5.75)	11004 (7.07)		344 (5.86)	10845 (7.59)	
Living arrangement			<0.001			0.001			0.052
Living with others	12370 (92.07)	272111 (91.13)		6973 (92.13)	141728 (91.01)		5397 (92.00)	130383 (91.27)	
Living alone	1065 (7.93)	26474 (8.87)		596 (7.87)	13999 (8.99)		469 (8.00)	12475 (8.73)	

### Demographic factors associated with depression: univariate findings

**Table 1** also reports depressive symptoms were significantly associated with age, marital status, nationality and eldercare status among both female and male participants. The depressed respondents with depressive symptoms were more often older, unmarried and with no eldercare for both genders. Among female participants, the depressed were more often living alone, whereas the male respondents with depressive symptoms tended to have lower educational level than those who were not reportedly depressed (**Table 1**).

### Health Ability factors associated with depression: univariate findings

**Table 2** shows distribution and prevalence of depressive symptoms by gender according to ADL, sensory and communication. Depressive symptoms were significantly associated with ADL, sensory and communication among both female and male participants. The depressed respondents with depressive symptoms were more with activities of daily living limitations and sensory and impaired communication ability for both genders.

**Table 2:** Distribution and prevalence of depressive symptoms by gender according to ADL and Sensory and communication.

Variable	Full Samples		P	Female		P	Male		P
	Not Depressed (n=13435)	Depressed (n=298585)		Not Depressed (n=7569)	Depressed (n=155727)		Not Depressed (n=5866)	Depressed (n=142858)	
ADL			<0.001			<0.001			<0.001
Unimpaired	8840	276800		4951	143685		3889	133115	
Impaired	4595	21785		2618	12042		1977	9743	
Sensory and communication			<0.001			<0.001			<0.001
Unimpaired	5438	262414		3096	136287		2342	126127	
Impaired	7997	36171		4473	19440		3524	16731	

### Binary logistic regression analysis of depressive symptoms: OR (95%CI)

**Table 3** reports the final logistical regression results. Regression findings suggest that geriatric depressive symptoms are significantly and positively associated with the lack of eldercare (OR=2.427, female; OR=1.426, male), living alone (OR= 1.430, female; OR= 1.179, male), ADL dysfunction (OR=1.528, female; OR=1.246, male), and impaired sensory and communication ability(OR=1.338, female; OR=1.185, male) among both female and male participants. Remarkably, geriatric depressive symptoms are only significantly associated with age (≥75, OR = 1.327), marital status (unmarried, OR=1.598), the number of children(no children, OR=2.271), and the living arrangement(living alone, OR= 1.430) among female participants.

**Table 3:** Logistics regression of ADL and Sensory and communication associated with depressive symptoms by gender.

Variable	Categories	Model 1, Female			Model 2, Male		
		0R	95%CI	P	0R	95%CI	P
Age(ref. 65-74)	≥75	1.327	1.088–1.671	0.005	1.074	0.864–1.325	0.515
Marital status(ref. Married)	Unmarried	1.598	0.908–1.697	0.021	1.020	0.634–1.537	0.924
Nationality(ref. Han)	Others	1.025	0.614–1.763	0.915	0.853	0.627–1.224	0.431
Education(ref. Middle school and above)	Elementary school and below	1.532	0.539–1.628	0.425	0.924	0.716–1.267	0.538
The number of children(ref. Have children)	No children	2.271	1.416–3.524	0.001	0.974	0.617–1.472	0.927
Eldercare status(ref. Have eldercare)	No eldercare	2.427	1.628–3.652	<0.001	1.426	1.057–1.913	0.016
Living arrangement(ref. Living with others)	Living alone	1.430	1.186–1.726	<0.001	1.179	0.996–1.395	0.055
ADL(ref. Unimpaired)	Impaired	1.528	1.184–1.978	0.002	1.246	1.034–1.626	0.024
Sensory and communication(ref. Unimpaired)	Impaired	1.338	1.024–1.761	0.035	1.185	0.931–1.537	0.041

### Regression coefficients across models: interaction effects

Test statistics of the interactions for gender shows significant gender differences in these associations were found for living alone, ADL dysfunction and impaired sensory and communication ability in **Table 4**. The results of this study provide evidence for the hypotheses that impaired ADL ability and sensory and communication abilities increase the likelihood of experiencing depressive symptoms in elderly individuals. These findings suggest that the impact of functional impairments on depressive symptoms differs based on gender and interventions targeting functional abilities could potentially alleviate depressive symptoms in the geriatric population.

**Table 4:** Regression coefficients across models: interaction effects.

Variable	Female vs. Male	
	χ2/t	p
Age	2.14	0.215
Marital status	0.01	0.937
Education	0.37	0.538
The number of children	0.89	0.329
Eldercare status	0.63	0.427
Living arrangement	6.31	0.017
ADL	5.72	0.021
Sensory and communication	7.15	0.014

## Discussion

### Key findings

Our study explored the gender differences in depressive symptoms among older adults in urban China, and further explored the effects of ADL, sensory and communication ability on depressive symptoms. The study shows a high prevalence of depressive symptoms was found in both genders, with a larger percentage in females, which is consistent with the results of previous studies. Females were found to be more often depressed than males. Approximately 95.69% of the participants had depressive symptoms according to the CESD-10, respectively, with male being 47.85% and female being 52.15%, which had no significant differences. Mental health professionals and researchers have long recognized the importance of understanding the gender differences in depression, particularly in the elderly population. Several studies have found that females are more likely to suffer from affective disturbances depression in later life than males. This gender difference is consistent across different countries, cultures, and ethnic backgrounds. According to previous studies, the prevalence of depression among women aged 65 and above is around two times higher than that among men in the same age group (22). The difference persists even after controlling for factors such as physical health, SES, and life events.

### Fertility status, living arrangement and depression

In our study, the number of children and living arrangement are statistically associated with depressive symptoms among female participants. In recent years, there has been an increasing interest in exploring the role of social factors, such as family structure and living arrangements, in the development and manifestation of late-life depression (23, 24). Several studies found that the number of children was associated with late-life depression in women but not in men. Specifically, having fewer children or no children was associated with a higher risk of depression in older women (25). The findings from previous researches suggest that the number of children may be an important risk factor for late-life depression in women. This may be due to the traditional role of women as caregivers and the social support provided by children. However, there is some evidence to suggest that the relationship between number of children and depression may be more complex and may vary by cultural and socioeconomic factors. The relationship between living arrangement and late-life depression was less consistent. Some studies found that living alone was a risk factor for depression in older adults, while others found no association. There was also some evidence to suggest that the relationship between living arrangement and depression may vary by gender (26). Additionally, the relationship between living arrangement and depression may be influenced by other social factors, such as social support, social isolation, and life events.

### Health ability and depression

We found that ADL impaired, sensory and communication impaired are all statistically associated with depressive symptoms among female and male participants, in which shows statistically significant gender difference. Depression can weaken an older adult’s motivation to perform ADL ability, such as dressing, bathing, and eating. As a result, routine tasks become more challenging, increasing the likelihood of functional impairment (23, 27). This impairment can further exacerbate depression, creating a vicious cycle that leads to a decline in quality of life. Several studies have examined the relationship between depression and ADL performance (28). One study found that elderly individuals with depression had more difficulty performing ADL tasks than those without depression. Additionally, a longitudinal study found that older adults with poorer ADL performance had a higher risk of developing depression over time (29, 30).

Changes in sensory and communication abilities can also contribute to the development of depression in older adults. The decline in sensory abilities, such as hearing and vision, can lead to social isolation and communication difficulties. These factors can increase the risk of depression, particularly in older adults who live alone or have limited social support (31). Several studies have investigated the relationship between sensory and communication abilities and depression in older adults (32, 33). One study reported that older adults with hearing loss were more likely to develop depression than those without hearing loss (34). Another study found that depression was associated with decreased visual acuity in older adults. Additionally, individuals with communication difficulties, such as language impairment or speech disorders, are at a higher risk for depression (35).

Although the relationship between depression and ADL and sensory and communication abilities is well-documented, it is unclear whether there are gender differences in this relationship. Some studies suggest that women are more vulnerable to the negative effects of functional impairment on depression. Other studies have reported that women experience higher levels of depression than men, regardless of their ADL or sensory and communication abilities (32–35). Gender differences in depression may also be influenced by sociocultural factors. Women are more likely to experience multiple roles and responsibilities, such as childcare and care-giving, which can increase the risk of depression. Additionally, women may be more likely to internalize negative thoughts and emotions, leading to a greater vulnerability to depression.

ADL and sensory and communication abilities play a significant role in the development of depression among older adults. Gender differences in depression and these abilities are complex and multifaceted, influenced by biological, psychological, and sociocultural factors. Further research is needed to better understand the mechanisms linking functional impairment and depression, and to develop interventions that can effectively address the needs of older adults with depression.

### Limitations

The main limitations includes the cross-sectional study design, the use of self-reported data, and without further clinical diagnosis for the respondents who met the screening criteria according to CES-D in the original research.

## Conclusions

Using the nationally representative data in China to explore the gender differences in geriatric depressive symptoms in urban China, with a particular focus on the role of activities of daily living (ADL) and sensory and communication abilities. The findings indicate that there are significant gender disparities in depressive symptoms among older adults, with women being more susceptible to depression. The study revealed that impaired ADL and sensory and communication abilities are associated with higher levels of depression in both genders. The study revealed that impaired ADL and sensory and communication abilities are associated with higher levels of depression in both genders. The research paper highlights the importance of considering these factors when developing interventions and support systems for the elderly population in urban China. By addressing impairments in ADL and sensory and communication abilities, mental health professionals can effectively target the underlying causes of depression and provide appropriate care to both men and women.

To address these gaps, future studies could focus on identifying the underlying biological, psychological, and sociocultural factors contributing to gender differences in geriatric depressive symptoms. This may involve examining the impact of hormonal changes, social roles and expectations, and cultural influences on mental health among the elderly in urban China. Moreover, considering the dynamic nature of gender roles and societal changes, future research should explore how cultural and social factors influence gender-specific experiences of depressive symptoms in urban China. This would enable healthcare professionals to tailor interventions that address the unique challenges faced by men and women in the context of evolving societal dynamics.

## Data availability statement

The original contributions presented in the study are included in the article/supplementary material. Further inquiries can be directed to the corresponding author. The baseline data collection was obtained from the West China Hospital of Sichuan University’s Biomedical Research Ethics Committee (2015-1184).

## Ethics statement

Ethical approval was not required for the study involving humans in accordance with the local legislation and institutional requirements. Written informed consent to participate in this study was not required from the participants or the participants’ legal guardians/next of kin in accordance with the national legislation and the institutional requirements.

## Author contributions

LG: Methodology, Software, Writing – original draft. MF: Conceptualization, Formal analysis, Writing – review & editing. LW: Writing – review & editing. LL: Writing – review & editing. CH: Writing – review & editing. XZ: Writing – review & editing. YL: Writing – review & editing. XH: Conceptualization, Visualization, Writing – review & editing.
